# Medical Fantasies

**Published:** 1856-02

**Authors:** 


### MEDICAL FANTASIES.

One of the earliest Hydropathic prescriptions we read of, was recorded long before the days of Preisnitz; it was given by an Ass to a brother Ass, was followed instanter, *to the death*, and has been kept up in the same style ever since. The legend goes in this wise:

Two Donkeys were travelling one hot summer's day, heavily laden, one with a sack of wool, the other with a sack of salt. Almost exhausted with heat and fatigue, they came at length to a river; and wisely enough, it was concluded that one should try the ford first. The one with the salt plunged in, and on reaching the opposite shore safely, found himself so much refreshed by the cooling of the waters, and so invigorated was he, that he felt all at once as if he had no load at all—as if he could carry two or three sacks more, and being naturally benevolent, he urged his companion to lose no time and plunge into the stream, triumphantly pleading his own delightful experience; so Assy No. two jumped in, *according to directions*, and—was crushed to the earth.

We scarcely need remind the reader, that in the first instance the salt was dissolved and passed down the stream, while the wool, absorbing more water, became more weighty, and hence the very signal failure of the prescription.

The wisest among men may learn a useful lesson from this homely fable. It is this reasoning a-la-Donkey, that fills the world with errors not only in medicine, but in morals; not merely errors in theory, but in practice; pervading every profession and every calling of human life. The mischief arises from *confounding* CAUSE *and* EFFECT *with* ANTECEDENCE *and* SUBSEQUENCE. If I faint and fall to the earth and cold water is thrown into my face, I "*come to;*" if spirits of hartshorn be applied to the nose, the same result is observed; hence these methods are resorted to, the world over, and the cold water and the hartshorn have the credit of restoration, but erroneously; they were applied, and the restoration followed; but this was merely antecedence and subsequence, the water was not the *cause* of the restoration, nor was the restoration the effect of the application of the water, for if a fainting man be laid upon his back, he will *come to* by simply being let alone, and in a much more gentle, gradual and agreeable way, without being shocked almost out of his senses or having his best clothes all drabbled over with water. The real cause of restoration is, *natural reaction*—it is a something which is kindly and wisely made a part of our being, by HIM whose ways to men are goodness and love personified; the name of this benign agency is beautifully denominated the *Vis Medicatrix Naturæ*, the power which nature has of curing herself. This is the doctor patronized by all regular physicians; but as no amount of argument would persuade the common people to do the same, we pass the point for the purpose of having a little fun at the expense of great men.

Taking a mere subsequence for an effect, the great *Martin Luther* declared, "If you run a stick through *three* frogs, dry them in the sun, and apply them to any pestilent tumor, they draw out all poison, and the malady will disappear." Suppose the frogs had been guillotined or hung, and then dried in the sun, it is not likely they would have been less efficacious. It requires some considerable time, especially in winter, to dry a frog, meanwhile the "pestilent tumor" would pass its crisis, and get well of itself. Modern wisdom has improved on Luther's prescription, for it has discovered that a chicken split open and applied while warm, is of sovereign efficacy in similar cases. The thing that cures is not the stuck frog, nor the divided pullet, but keeping the parts soothingly moist and warm for some time, without disturbance. A poultice made of flaxseed or bread and milk, would have all the virtues of the frog or the chicken, with the no small advantage of being more instantly available. It would require some considerable hunting to secure three frogs in New York, or any where in mid winter, and as for our chickens, they are all dead a long time ago, long enough to grow very tender.

The great Bishop Berkeley, one of the most accomplished and best educated men of the age in which he lived, wrote a book "*concerning the virtues of* TAR WATER," advocating its efficacy in coughs, colds and consumption, dropsies, fevers and small pox. Some people made fun of the Bishop, but he confidently appealed to time and observation. But time is a slow coach for the Bishop, as a hundred and ten years have failed to certify his theory. One day the Bishop was taken suddenly ill, but he hadn't a bit of Tar in his house, and before any could be had, he——died. It was a great oversight that, not to have had two or three barrels of Tar stowed away in his house to meet emergencies. Bacon believed that the application of ointment to a weapon which inflicted a wound, was more efficacious than if it were applied to the wound itself; and the great Boyle believed that the thigh bone of a criminal who had suffered death, was a cure for some bowel affections, which indeed is a fact, with this limitation, any other bone of any other man, brute or beast, if burned and pulverized, would have been equally efficacious; quite as efficacious as a remedy once uttered in our hearing: "A chicken's gizzard well boiled, then burnt to a cinder, then finely pulverized, and swallowed; a cure for the diarrhœa." And so it is in some forms; but burnt cork is equally efficacious; and it is quite likely, in fact certain, that a tablespoonful of tad-poles or shrimps, or a good big craw-fish, burned to a cinder, then pulverized, would avail as much. But instead of regarding these *outrè* articles as having medicinal merits, or being the cause of cure, we should endeavor to ascertain whether there was not some one quailty common to all, and whether there was not reason to believe that all the virtue resided in that one quality. At the first glance we perceive that *innocuous, impalpable fineness*, is the great requisite; hence, in certain forms and stages of loose bowels we find that the nitrate of bismuth, or a tablespoon of fine flour, stirred in a little cold water, and drank quickly, are both very reliable remedies; but let no reader illustrate his genealogy by running to the flour barrel the next time he has a loose visitation, for if it be a *bilious* diarrhœa, it will do no good; if it be the premonitory of *cholera*, the delay might be death; or if it be the looseness of a surfeit, the flour would have no effect; in either of these cases, show yourself a sensible man, by lying down and sending for your family physician.

The great lesson we desire to inculcate in this article is—*If you would avoid serious errors, do not confound mere* SUBSEQUENTS *with* CAUSES *in your philosophy;* such a mistake is the rock on which millions have wrecked all human hopes, and millions more will do the same, but among them, we trust, none of our readers will be found.

